# Transcranial Direct Current Stimulation Combined with Aerobic Exercise to Optimize Analgesic Responses in Fibromyalgia: A Randomized Placebo-Controlled Clinical Trial

**DOI:** 10.3389/fnhum.2016.00068

**Published:** 2016-03-10

**Authors:** Mariana E. Mendonca, Marcel Simis, Luanda C. Grecco, Linamara R. Battistella, Abrahão F. Baptista, Felipe Fregni

**Affiliations:** ^1^Department of Neuroscience and Behavior, Institute of Psychology, University of São PauloSão Paulo, Brazil; ^2^Physical and Rehabilitation Medicine Institute of the University of São Paulo, Medical School General HospitalSão Paulo, Brazil; ^3^Pediatric Neurosurgical Center - Rehabilitation (CENEPE)São Paulo, Brazil; ^4^Laboratory of Neuromodulation, Department of Physical Medicine and Rehabilitation, Harvard Medical School, Spaulding Rehabilitation Hospital and Massachusetts General HospitalBoston, MA, USA; ^5^Laboratory of Functional Electrostimulation, Department of Biomorphology, Federal University of BahiaBahia, Brazil

**Keywords:** transcranial direct current stimulation (tDCS), fibromyalgia, aerobic exercise, combined therapy, motor cortex

## Abstract

Fibromyalgia is a chronic pain syndrome that is associated with maladaptive plasticity in neural central circuits. One of the neural circuits that are involved in pain in fibromyalgia is the primary motor cortex. We tested a combination intervention that aimed to modulate the motor system: transcranial direct current stimulation (tDCS) of the primary motor cortex (M1) and aerobic exercise (AE). In this phase II, sham-controlled randomized clinical trial, 45 subjects were assigned to 1 of 3 groups: tDCS + AE, AE only, and tDCS only. The following outcomes were assessed: intensity of pain, level of anxiety, quality of life, mood, pressure pain threshold, and cortical plasticity, as indexed by transcranial magnetic stimulation. There was a significant effect for the group-time interaction for intensity of pain, demonstrating that tDCS/AE was superior to AE [*F*_(13, 364)_ = 2.25, *p* = 0.007] and tDCS [*F*_(13, 364)_ = 2.33, *p* = 0.0056] alone. *Post-hoc* adjusted analysis showed a difference between tDCS/AE and tDCS group after the first week of stimulation and after 1 month intervention period (*p* = 0.02 and *p* = 0.03, respectively). Further, after treatment there was a significant difference between groups in anxiety and mood levels. The combination treatment effected the greatest response. The three groups had no differences regarding responses in motor cortex plasticity, as assessed by TMS. The combination of tDCS with aerobic exercise is superior compared with each individual intervention (cohen's *d* effect sizes > 0.55). The combination intervention had a significant effect on pain, anxiety and mood. Based on the similar effects on cortical plasticity outcomes, the combination intervention might have affected other neural circuits, such as those that control the affective-emotional aspects of pain.

Trial registration: (www.ClinicalTrials.gov), identifier NTC02358902.

## Introduction

Fibromyalgia is a chronic pain syndrome that is characterized by the presence of diffuse pain throughout the body and secondary symptoms, such as sleep disturbances and cognitive dysfunction (Bernardy et al., 2013). The etiology of fibromyalgia is unknown, but its onset is attributed to the continuity of painful stimuli, triggering mechanisms of central sensitization (Cagnie et al., 2014). These processes lead to maladaptive plastic changes in cortical activity in various regions, including the classical areas of the pain neuromatrix circuit and other neural circuits, such as the primary motor cortex.

In this context, the Motor Cortex (M1) is an important area to understand the pathophysiology and treatment of Fibromyalgia Sindrome (FMS). A recent review noted that many studies in other pain syndromes reported increased activation in this region to rest and increased response to nociceptive sensory stimuli, demonstrating its interaction with other areas of pain modulation (Castillo Saavedra et al., 2014). Current studies are using neuromodulation techniques to modify the excitability of the M1 and provide relief from the symptoms of chronic pain (Fregni et al., 2006; Valle et al., 2009; Mori et al., 2010; Mendonca et al., 2011; DaSilva et al., 2012; Yoon et al., 2013).

One such technique is Transcranial Direct Current Stimulation (tDCS). tDCS promotes the modulation of brain activity by subtly altering the excitability of the neuronal membrane, and its prolonged and continuous application can effect plastic modification, with activation of NMDA receptors and the Long Term Potentiation (LTP) phenomenon (Nitsche et al., 2003; Fritsch et al., 2010; Monte-Silva et al., 2013).

Preliminary trials that have tested tDCS of the M1 in reducing fibromyalgia pain have reported positive results, although the effects varied and, in some cases, were small (Fregni et al., 2006; Valle et al., 2009; Mendonca et al., 2011). Based on the mechanisms of tDCS, one approach to optimizing its effects is to combine it with a behavioral intervention that promotes activation in the same neural circuit. Thus, we hypothesize that tDCS of the M1, combined with aerobic exercise, would enhance the effects of tDCS on FMS pain.

Aerobic exercise acts systemically, influencing various aspects of body function. For example, it can affect a large neural circuit via afferent input (bottom-up) from somatosensory stimulation and a neuroendocrine response (Schwarz and Kindermann, 1992; Goldfarb and Jamurtas, 1997; Kramer and Erickson, 2007). This technique has long-lasting effects and can be sustained by the patient to maintain the improvement (Colcombe et al., 2004).

We tested the clinical and neurophysiological effects of the combination of tDCS and aerobic exercise on a treadmill over 1 month to generate results of a new intervention and to understand how modulation of the M1 circuit leads to pain control. Our main aim was to assess whether the combined intervention of tDCS and aerobic exercise would induce significantly greater pain reduction as compared to tDCS alone and aerobic exercise alone.

## Methods

### Participants

Participants were recruited through social networks, local health care facilities, and referrals for a waiting list for treatment at the Institute of Physical Medicine and Rehabilitation, Faculty of Medicine, University of São Paulo, Brazil. The study population comprised individuals who were diagnosed with fibromyalgia. The diagnosis was performed by medical specialists taking into consideration the modified criteria from ACR (Wolfe et al., 2011). For the evaluations were taken into account the terms described by the modified evaluation criteria and who fulfilled the following eligibility criteria: (a) completed high school and (b) age between 18 and 65 years. Subjects were excluded if they: (a) were on medication for pain control for less than 2 months; (b) had been treated for depression for less than two months; (c) had epilepsy, psychiatric disorders, or any recent episode of neurological disorders, such as idiopathic syncope; (d) were pregnant and infant-aged; (e) had metallic implants in the brain; (f) were using illicit drugs; or (g) had been undergoing some type of physical treatment for less than 2 months.

All patients signed informed consent forms prior to initiation of the study procedures. This research was approved by the research ethics committee at CONEP under registration number CAAE 08603612.0.0000.5511. The trial was also registered at clinicaltrials.gov (identifier NTC02358902).

### Experimental design

The design was a clinical, randomized, double-blind study with 2 months of follow-up. Data were collected from January 2013 to November 2014. Recruitment was performed during the entire period since interventions were carried out in group each month.

**Figure 1:**
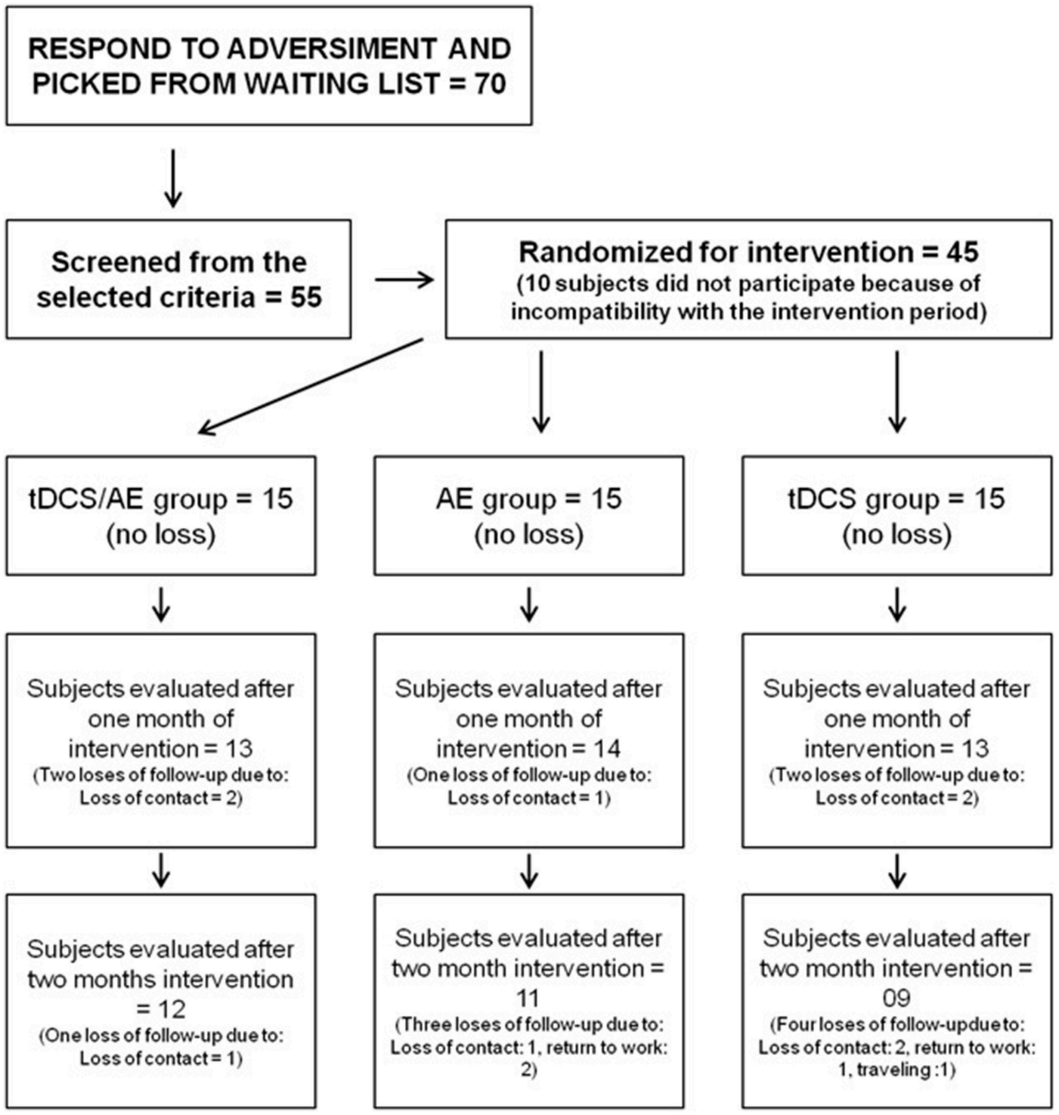
**Research flowchart**.

A total of 45 participants were included (Figure 1). Randomization was performed by a blinded therapist using sealed envelopes for each individual. The subjects were divided into 3 intervention groups: **tDCS/AE**, which received active intervention of aerobic exercise training and active tDCS intervention; **AE**, which received active intervention of aerobic exercise and placebo tDCS; and **tDCS**, which received placebo AE and active intervention for tDCS.

Participants were blinded to the intervention groups, as were the therapists who performed the evaluation.

### Outcomes

All variables were measured 1 week before the beginning of the intervention (baseline), after intervention period (T2) and during the periods of follow-up conducted 1 month (T3) and 2 months (T4) after the end of the intervention period. For variables such as pain intensity and intensity of anxiety, these evaluations were performed every day before the intervention. The assessment of cortical excitability was conducted at baseline, T2, T3, and T4 and after the fifth day of intervention (first week) (T1), which corresponding to the end of the stimulation period. This strategy was chosen to minimize long periods of evaluation during the procedure, reducing burden to subjects.

### Primary outcome

The Visual Numeric Scale (VNS) was used to assess the intensity of pain, as reported by the patient. This straight 10-cm scale is numbered from 0 to 10, in which 0 represents no pain and 10 is the most pain imaginable. Subjects were asked to mark the number that best reflected the symptoms of pain at that moment.

### Secondary outcomes

#### Anxiety levels

Anxiety levels were measured using the VNS for anxiety (from 0 to 10). Also for this outcome, assessments was carried out at baseline, every day before the intervention, post intervention (T2) and follow-up periods (T3, T4).

#### Pressure pain threshold

Pressure pain threshold (PPT) was evaluated with a pressure algometer (Wagner Instruments, USA) to establish the minimum pressure that triggered the pain at the thenar region of the hand and the uppermost portion of the anterior tibialis. These areas were chosen to determine the systemic effects of the interventions. For the statistical analysis, the average of these values was calculated.

#### Quality of live

Quality of life was assessed using the SF-36 quality of life questionnaire for all subscales: vitality, physical functioning, bodily pain, general health perceptions, physical role functioning, emotional role functioning, social role functioning, and mental health.

#### Mood

The Beck Depression Inventory was also used to measured symptoms of depression.

#### Cortical excitability

Cortical excitability was examined by Transcranial Magnetic Stimulation (TMS) using a figure-of-eight magnetic stimulator coil (BiStim^2^ Magstim, UK). Responses to stimuli that were applied to the motor cortex were recorded in the adductor muscle of the thumb of the contralateral hand. The responses of the motor evoked potential (MEP) were amplified and filtered by surface electromyography (Micromed SpA, Italy). The signals were then transferred to a personal computer for offline analysis using software for data collection (SystemPlus Evolution, Micromed SpA, Italy).

Motor threshold, motor evoked potential, intracortical inhibition, and intracortical facilitation were measured (Kobayashi and Pascual-Leone, 2003). All measures were performed at the left M1 in which the tDCS was held. Motor threshold and motor evoked potentials were evaluated by single-pulse TMS. Motor threshold was found using the lowest intensity for the TMS pulse over the M1 capable to generate a peripheral response of at least 50 microvolts of amplitude at the electromyography. The same technique was used to determine the MEP at 120% of the intensity found for the motor threshold. Ten MEPs were measured at each stage.

Intracortical inhibition (ICI) and intracortical facilitation (ICF) were evaluated by paired-pulse technique. For ICF, a conditional pulse with an intensity of 80% of the motor threshold and a test pulse with the MEP intensity were used. The interstimulus interval was 10 ms for ICF. In measuring intracortical inhibition, the same parameters for the conditional and test stimuli and an interstimulus interval of 2 ms were used. In each individual, 15 measures of ICF and ICI each were made, randomized between inhibition, facilitation, and MEP, totaling 45 pulses for this step.

#### Adverse effects

A questionnaire on the adverse effects of tDCS was given to evaluate the adverse effects of transcranial direct current stimulation.

To evaluate the adverse effects of AE, we recorded any musculoskeletal symptoms—such as pain, fatigue, tingling—or cardiovascular symptoms—such as shortness of breath, chest pain, exorbitant increased blood pressure—every day of intervention.

#### Interventions

**Figure 2:**
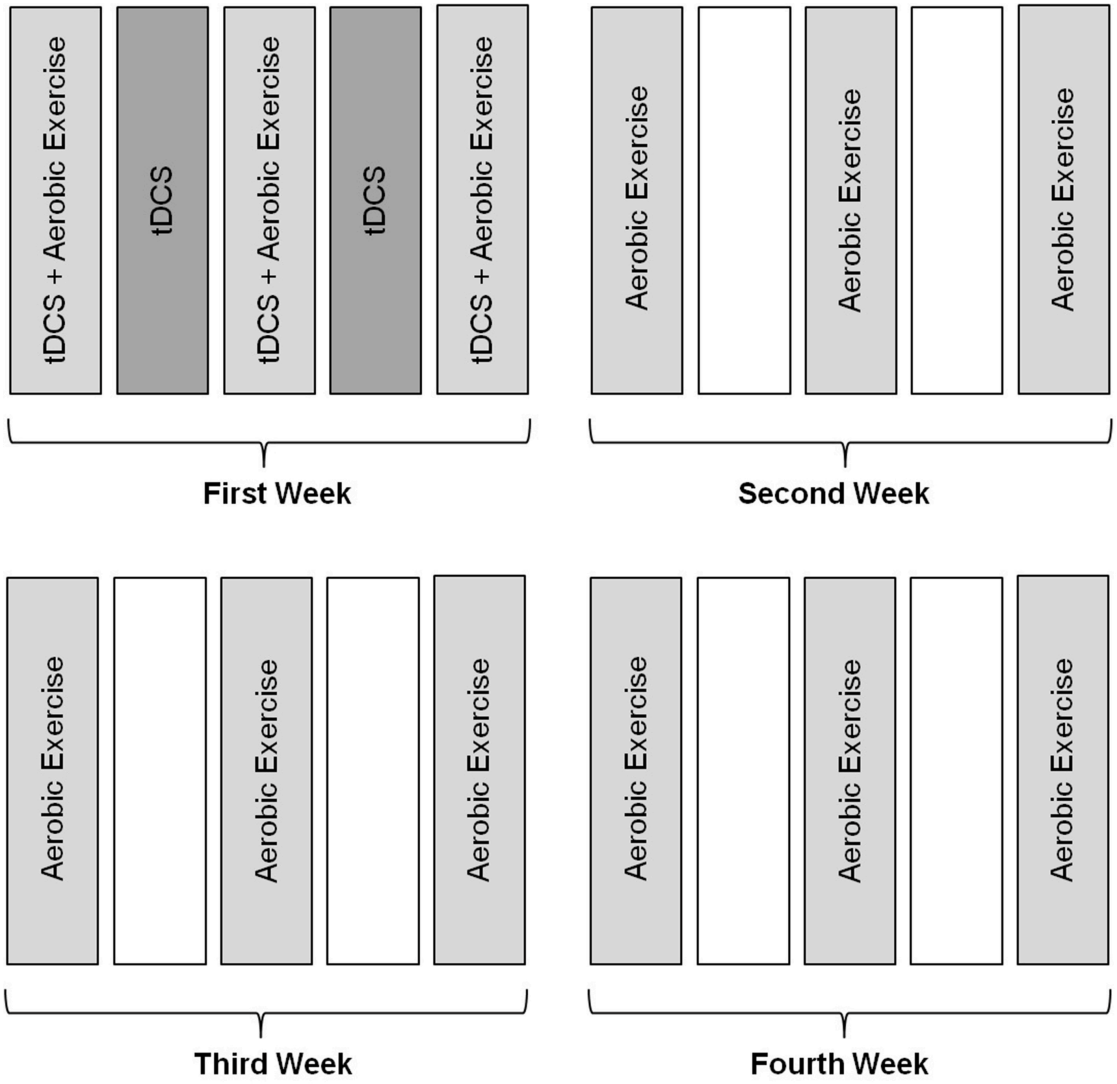
**Methodology of intervention**. Subjects received intervention with tDCS during the first week for five consecutive days, associated with aerobic exercise training performed three times a week for a month.

**Figure 3:**
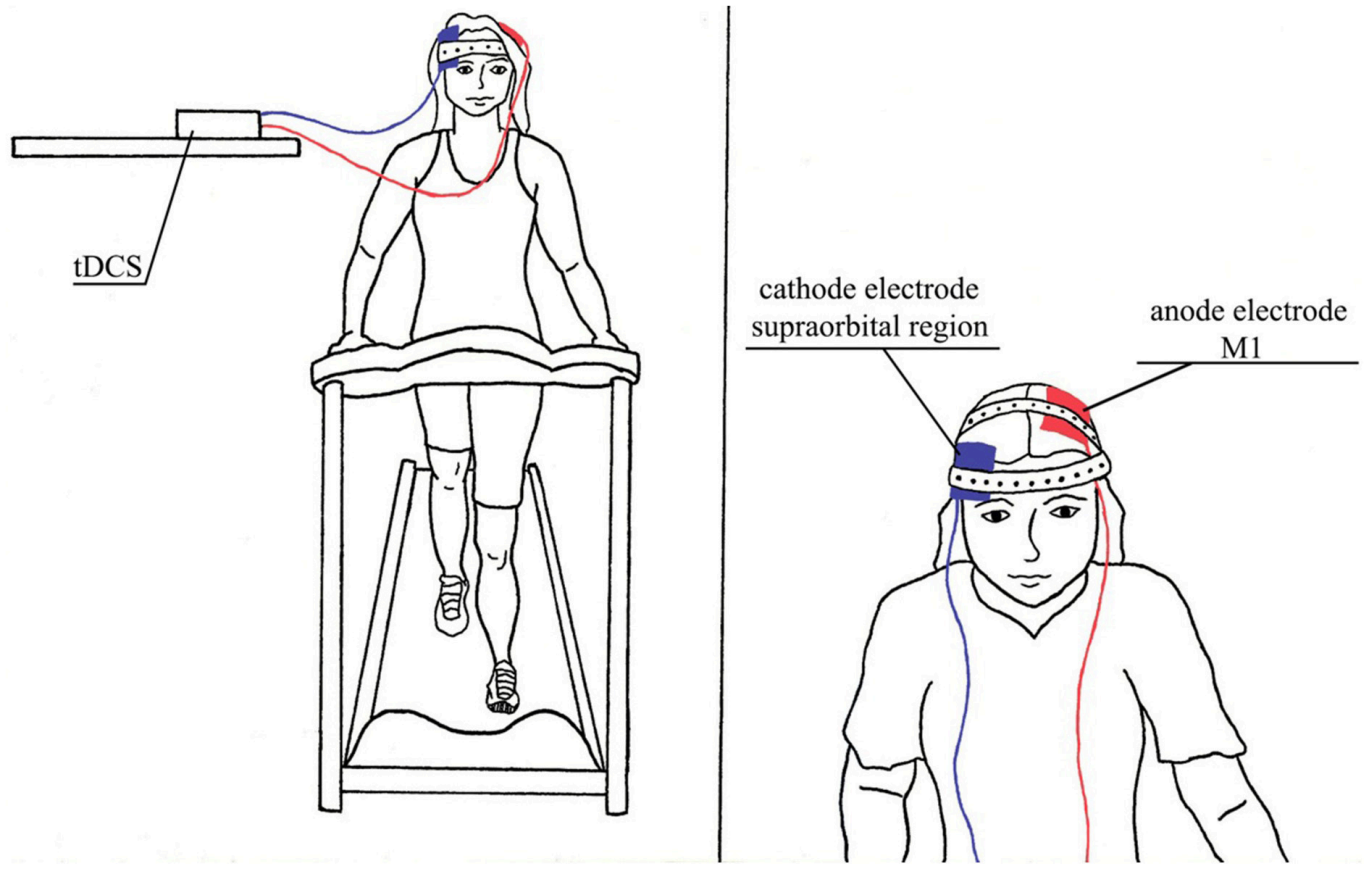
**Illustration of interventions in combination, tDCS/AE group which was performed aerobic exercise in combination with tDCS**.

The treatment was administered for 4 weeks. During the first week, the subject underwent tDCS every day (Monday to Friday) and aerobic exercise 3 days per week (neuromodulatory phase). On the days on which exercise was performed, the 2 techniques were executed in combination simultaneously. In the following weeks, the subject attended to perform the procedure on 3 days per week for aerobic exercise only (Figures 2, 3).

Standard safety assessments were performed by the nursing team before and after every visit day including heart and respiratory rate and blood pressure.

#### Intervention 1: transcranial direct current stimulation

tDCS was performed using a monophasic current device (DC stimulator, NeuroCom, Germany). Pairs of silicon sponge surface electrodes (35 cm^2^) were soaked in saline and positioned as follows: the anode was placed over the region of the primary motor cortex (M1) per the International 10/20 system at point C3 (M1 left), and the cathode was placed over the supraorbital region, contralateral to the anode (right).

The treatment method entailed 5 days of stimulation with monophasic continuous current with an intensity of 2 mA for 20 min. For stimulation a gradual current ramp-up and ramp-down with 30 s duration was used.

The sham procedure for tDCS was performed with same placement of electrodes as in the active group, but the stimulation was administered for only the initial 30 s, with the power turned off for the remaining period.

#### Intervention 2: aerobic exercise

Aerobic exercise was performed on a treadmill (Kikos E100, Brazil) for a period of 30 min per session. The exercise was scheduled to start at an intensity of 60% of the maximum Heart Rate (HR) for each patient. Maximum HR was defined as HRmax = 208 − (0.7 * age; Tanaka et al., 2001). HR was monitored throughout the entire procedure (heart monitor, Oregon Scientific, Brazil). After the second week, the intensity could be increased to 70% of the maximum HR, based on the individual's response. At the beginning and end of the exercise, the lower limbs were stretched in each session.

The sham procedure for AE consisted of subjects undergoing the training on the treadmill, but HR was maintained within 5% of the resting HR at the minimum speed on the treadmill.

#### Statistical analysis

All subjects completed the intervention period and carried out the post intervention assessment (T2). There was a loss of 12% of the sample in the first follow-up, and a loss of 28% of the sample in the second period of follow-up. Dropouts during follow-up were similar across groups. Specific missing data per group is described in Figure 1. Missing data were treated by intention-to-treat analysis, taking into account the method of the last observation carried forward. Sensitivity analysis was carried out with complete cases analysis. The Kolmogorov-Smirnov test demonstrated normal distribution of the data. Thus, parametric tests were performed, and the data were expressed as mean and standard deviation for the analysis and as mean and standard error in the graphs.

To compare the effects of tDCS and aerobic exercises on the main outcome variable—the VNS—we used mixed ANOVA, including the main effects of time [baseline, each day before the intervention (Days 1–13) and the follow-up period (1 and 2 months)], group (tDCS/AE, AE, and tDCS), and the interaction group X time. *Post-hoc* analyses were conducted using reduced ANCOVA models (for each time point: T1, T2, T3, and T4) adjusted for variables indexing baseline psychiatric and pain characteristics since these variables have an influence on final pain symptoms.

For other outcome variables, as dependent variables in the ANOVA models, we used the SF36 (all subscales), Beck Depression Inventory, pressure pain threshold, and neurophysiological parameters.

The independent fixed variables were time (baseline, post-treatment, follow-up 1, and follow-up 2), group (tDCS/AE, AE, and tDCS), and the group-treatment interaction. The effect size (cohen's *d* effect size) was calculated from the difference in values between baseline and post-treatment comparing the combination group with the other groups.

A similar analysis was conducted for the secondary outcomes. The predictors of outcome were analyzed by linear regression using univariate models, with the difference in pain intensity before and after the intervention as the dependent variable and age, time of pain, VNS values at baseline, SF36 (all subscales), Beck Depression Inventory, and changes in neurophysiological parameters at the post-treatment evaluation as independent variables. A *p* < 0.05 indicated a statistically significant result. The data were organized and tabulated using Stata 12.

## Results

Of the study participants, 44 were female. Considering the total sample all were right-handed, with a mean age of 47.4 (±12.1), and mean duration of pain of 138.5 (±94.2) months. Other demographic data are available in Table 1. Forty five individuals completed the intervention period. For the follow-up period there were three losses in group tDCS/AE, four losses in group AE, and six losses in group tDCS (Figure 1).

**Table 1:** **Sample data at baseline**.

	**tDCS + AE**	**AE**	**tDCS**	*p*-value^*^
Age (years) (±SD)	44.5 (±14)	48 (±11.8)	49.9 (±10.6)	0.4
Gender (F/M)	14/1	15/0	15/0	
Regular exercises (Y/ N)	2/13	4/11	3/12	
Pain duration (months) (±SD)	140.6 (±72.2)	149.3 (±111.1)	125.6 (±100.2)	0.7
Hours of sleep (±SD)	5.3 (±1.5)	5.8 (±1.5)	5.7 (±1.8)	0.7
VNS (±SD)	7.3 (±1.7)	6.8 (±2.0)	7.2 (±1.2)	0.72

* *Data are presented as mean and standard deviation. Analyzes performed by one way ANOVA*.

### Primary outcome: visual numeric scale

**Figure 4:**
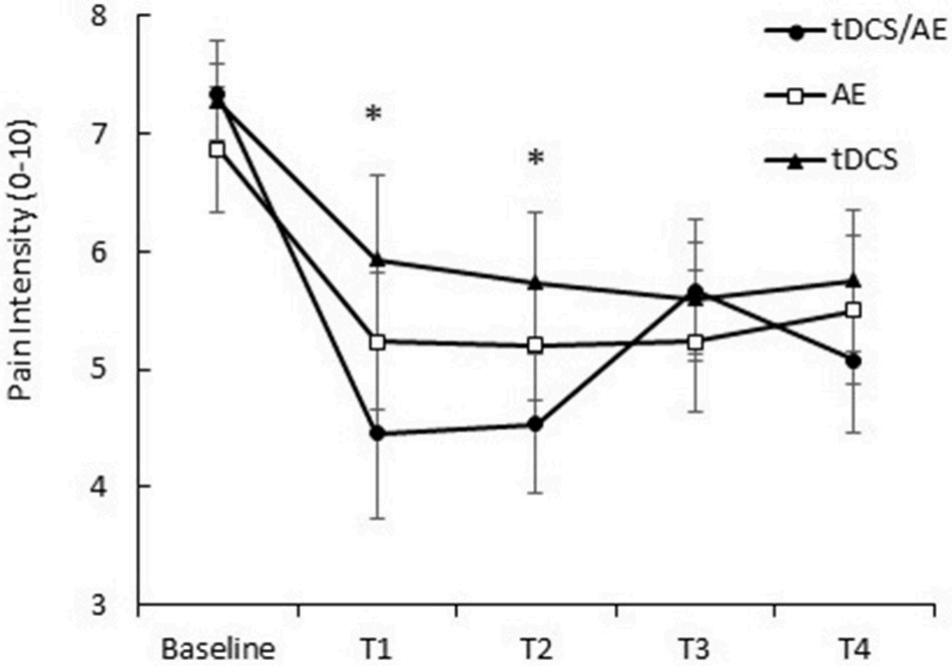
**Response of pain intensity (VNS pain)**. T1, assessment after the fifth day of intervention; T2, assessment after 1 month of intervention; T3, assessment after 1 month of the end of the intervention (follow-up 1); T4, assessment after 2 months of the end of the intervention (follow-up 2). Data presented as mean and standard error. ^*^Statistical analysis demonstrated significant result for T1 (*p* = 0.02) and at T2 (*p* = 0.03) between tDCS/AE group and tDCS group.

Pain intensity had a significantly effect on interaction time vs. group [*F*_(26, 546)_ = 2.08, *p* = 0.0015]. Similarly, there were significant main effects of group [*F*_(13, 546)_ = 6.78, *p* < 0.001] and time [*F*_(2, 546)_ = 32.16, *p* < 0.001]. By *post-hoc* analysis, there was a difference between the tDCS/AE and AE groups [*F*_(13, 364)_ = 2.25, *p* = 0.007] and the tDCS/AE and tDCS groups [*F*_(13, 364)_ = 2.33, *p* = 0.0056]. Analysis using covariate adjustment—with baseline psychiatric (anxiety level and mental health—SF-36) and pain characteristics—showed that there are significant changes at day 5 (end of stimulation—T1) and at the end of the protocol (T2) (*p* = 0.029 and *p* = 0.030, respectively), but not at the two follow-ups (*p* > 0.5 for both analyses) (T3 and T4) (Figure 4). Values of mean, standard deviation and percentage of improvement are described in Table 2.

**Table 2:** **Mean and standard deviation values of primary outcome (VNS-pain)**.

	**tDCS/AE group**	**AE group**	**tDCS group**
**BASELINE**			
Mean (±SD)	7.3 (±1.75)	6.8 (±2.0)	7.2 (±1.27)
**T1**			
Mean (±SD) % of improvement from baseline	4.4 (±2.85) 39.7%	5.2 (±2.25) 23.5%	5.9 (±1.27) 18%
**T2**			
Mean (±SD) % of improvement from baseline	4.5 (±2.29) 38.3%	5.2 (±1.83) 23.5%	5.7 (±2.31) 20.8%
**T3**			
Mean (±SD) % of improvement from baseline	5.6 (±2.31) 23.2%	5.3 (±2.32) 22.0%	5.5 (±1.91) 23.6%
**T4**			
Mean (±SD) % of improvement from baseline	5.0 (±2.4) 31.5%	5.5 (±2.45) 19.1%	5.7 (±2.38) 20.8%

Subsequent analysis using covariate adjustments demonstrated a difference between the groups tDCS/AE and tDCS at the end of the first week of intervention (effect size = 0.6, *p* = 0.02) and at the end of the 1 month intervention (effect size = 0.56, *p* = 0.03). For the comparison between the groups tDCS/AE and AE, although effect sizes were also large, there was no significant differences at day 5 (effect size = 0.68, *p* = 0.14) and, at the end of the 1 month intervention, although *p*-value was less than 0.1, it did not reach significance (effect size = 0.59, *p* = 0.08). The comparisons between the groups AE and tDCS revealed no significant differences (*p* > 0.5 for the comparisons between day 5 and end of 1 month). In fact the effect sizes comparing these two groups were very small (effect size day 5 = 0.12 and effect size at end of month = 0.07).

Sensitivity analysis demonstrated no difference in statistical results.

### Secondary outcomes

#### Anxiety level

**Figure 5:**
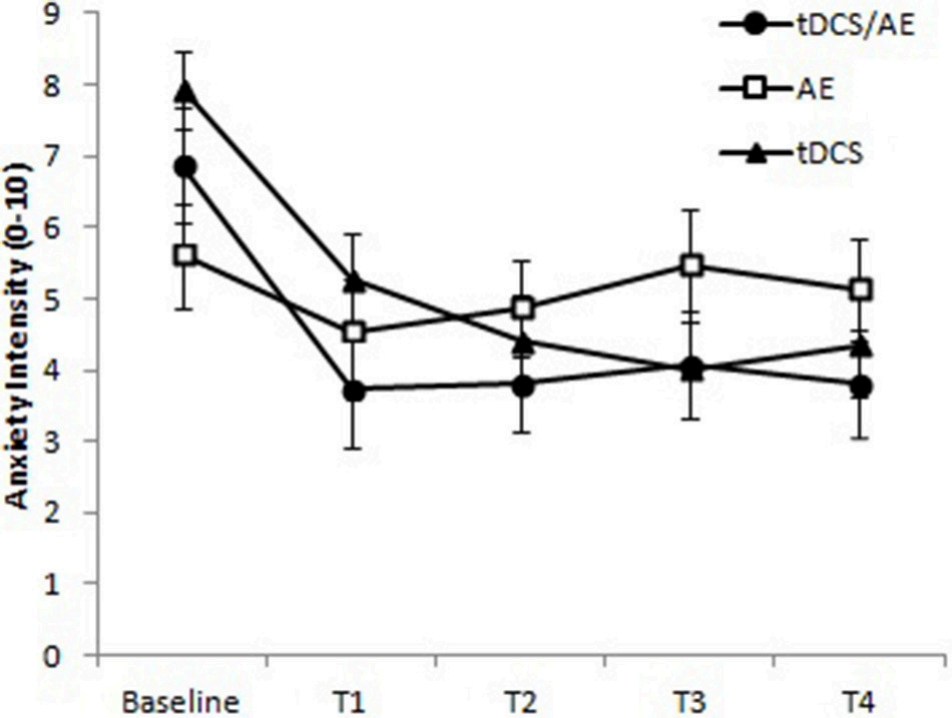
**Response for level of anxiety (VNS anxiety)**. T1, assessment after the fifth day of intervention; T2, assessment after 1 month of intervention; T3, assessment after 1 month of the end of the intervention (follow-up 1); T4, assessment after 2 months of the end of the intervention (follow-up 2). Paired evaluation between groups *p* < 0.001. Data presented as mean and standard error.

Anxiety level showed a significant result for the time-group interaction [*F*_(8, 168)_ = 3.86 *p* < 0.001] and time [*F*_(4, 168)_ = 11.70, *p* < 0.001] but not for group [*F*_(42, 168)_ = 7.17, *p* = 0.09; Figure 5).

#### Pressure pain threshold

**Figure 6:**
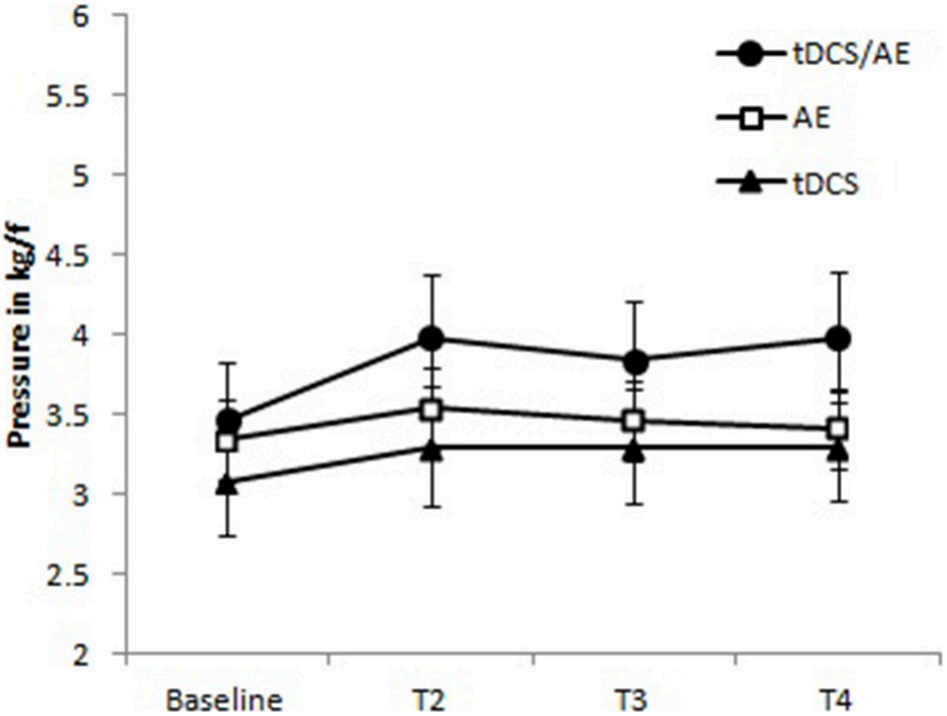
**Data for pressure pain threshold**. Group tDCS/AE demonstrating a relative of the pressure pain threshold increased, maintained during periods of follow-up. Group AE slight increase after the intervention, apparently not maintained at follow-up period. tDCS group with mild increase in the pressure pain threshold, being held in the follow-up period. No statistical significant data were observed. Data shown as mean and standard error.

With regard to pressure pain threshold, the time-group interaction was not significant [ANOVA, *F*_(9, 126)_ = 2.78, *p* = 0.08]. but because the *p*-value of this interaction was less than 0.1, we also calculated the main effects and found that group [*F*_(3, 126)_ = 4.44, *p* = 0.005] and time [*F*_(2, 126)_ = 77.87, *p* < 0.001] were significant. The results are shown in Figure 6.

#### Quality of life: SF-36

For the vitality, physical functioning, bodily pain, physical role functioning, emotional role functioning, social role functioning, and mental health subscales, no significant differences for the main interaction between time and group were observed (*p* > 0.05 for all). The data on the mean and standard deviations are listed in Table 3.

**Table 3:** **Mean and standard deviation for SF-36 questionnaire and Beck depression inventory results**.

	**tDCS/AE group**				**AE group**				**tDCS group**			
	**Baseline**	**T2**	**T3**	**T4**	**Baseline**	**T2**	**T3**	**T4**	**Baseline**	**T2**	**T3**	**T4**
Physical functioning	41.0(4.8)	48.3(4.0)	49.0(3.9)	48.0(4.5)	45.0(4.1)	49.3(4.8)	53.0(5.2)	50.6(4.9)	37.6(5.0)	41.0(5.8)	42.69(5.6)	45.3(6.0)
Physical role functioning	18.3(7.5)	36.2(9.0)	41.2(8.3)	52.8(10.3)	20.0(7.7)	36.6(10.3)	35.0(10.0)	40.0(10.0)	16.6(7.1)	26.6(9.9)	23.3(9.2)	30.0(10.4)
Bodily pain	25.6(4.6)	44.3(4.5)	43.2(4.3)	49.2(5.4)	27.1(3.0)	40.4(4.1)	39.6(3.8)	40.4(4.1)	21.6(2.6)	30.6(3.7)	29.2(3.7)	30.6(3.3)
General health perceptions	36.3(4.4)	42.8(3.3)	41.4(4.2)	42.1(3.4)	38.2(5.0)	47.5(5.1)	44.9(4.1)	43.9(4.3)	36.0(4.2)	37.1(4.5)	39.8(3.8)	38.1(3.8)
Vitality	30.0(4.7)	39.6(4.2)	43.3(4.6)	40.3(5.0)	39.0(5.4)	48.6(4.0)	47.0(4.2)	49.6(4.2)	24.6(4.1)	31.6(5.4)	32.6(5.3)	31.0(4.8)
Social role functioning	58.8(7.7)	62.7(7.0)	67.2(7.5)	73.5(7.4)	50.8(6.3)	58.3(6.5)	57.5(7.0)	60.7(7.0)	29.9(5.4)	34.6(6.7)	35.5(6.0)	37.1(7.0)
Emotional role functioning	47.4(12.3)	66.6(10.7)	66.6(11.4)	69.6(10.5)	36.0(11.2)	81.3(8.9)	76.0(8.4)	66.5(9.0)	22.2(10.6)	25.1(10.4)	29.5(10.1)	33.9(10.1)
Mental health	42.1(5.1)	57.1(6.4)	54.6(5.5)	53.6(5.8)	46.6(5.9)	56.0(6.3)	53.8(6.0)	53.8(6.1)	32.5(5.8)	41.3(6.2)	43.2(6.0)	48.6(6.6)
Beck depression inventory	20.8(2.1)	13.8(2.1)	14.1(2.1)	13.6(2.4)	21.0(3.1)	18.5(3.2)	16.2(3.2)	16.8(3.4)	25.0(2.7)	20.2(3.0)	19.0(3.0)	19.2(3.6)

*Values are represented as mean (± standard deviation)*.

For the general heath perceptions subscale, there was a significant result for the time-group interaction [*F*_(6, 84)_ = 3.9, *p* = 0.001] and for group [*F*_(3, 4)_ = 7.4, *p* = 0.004], but no significant differences were noted in the effect of time [*F*_(2, 2)_ = 0.6, *p* = 0.549]. By *post hoc* analysis, there was a difference before the intervention for the combination group vs. the AE and tDCS groups (*p* = 0.003 and *p* = 0.012, respectively) at the end of intervention (T2) (AE vs. tDCS *p* = 0.009) and at follow-up 2 (T4) (tDCS/AE vs. tDCS *p* = 0.006).

#### Mood

The evaluation of Beck Depression Inventory scores demonstrated no statistical significance for the interaction of time and group [*F*_(6, 123)_ = 0.84, *p* = 0.54], but group [*F*_(2, 123)_ = 8.55, *p* < 0.001] and time [*F*_(3, 123)_ = 18,26, *p* < 0.001] had significant effects. In an exploratory analysis of this variable, removing the follow-up periods to better understand the immediate effects of the interventions, significant results were found for the time-group interaction [*F*_(2, 41)_ = 3.22, *p* = 0.05], group [*F*_(2, 41)_ = 6.22, *p* = 0.004], and time [*F*_(1, 41)_ = 37.33, *p* < 0.00]—the combined intervention group experienced the largest decrease in depression intensity (*p* = 0.001) vs. the AE group at the end of the intervention period (T2). Data of mean and standard deviation described in Table 2.

#### Neurophysiological data

**Figure 7:**
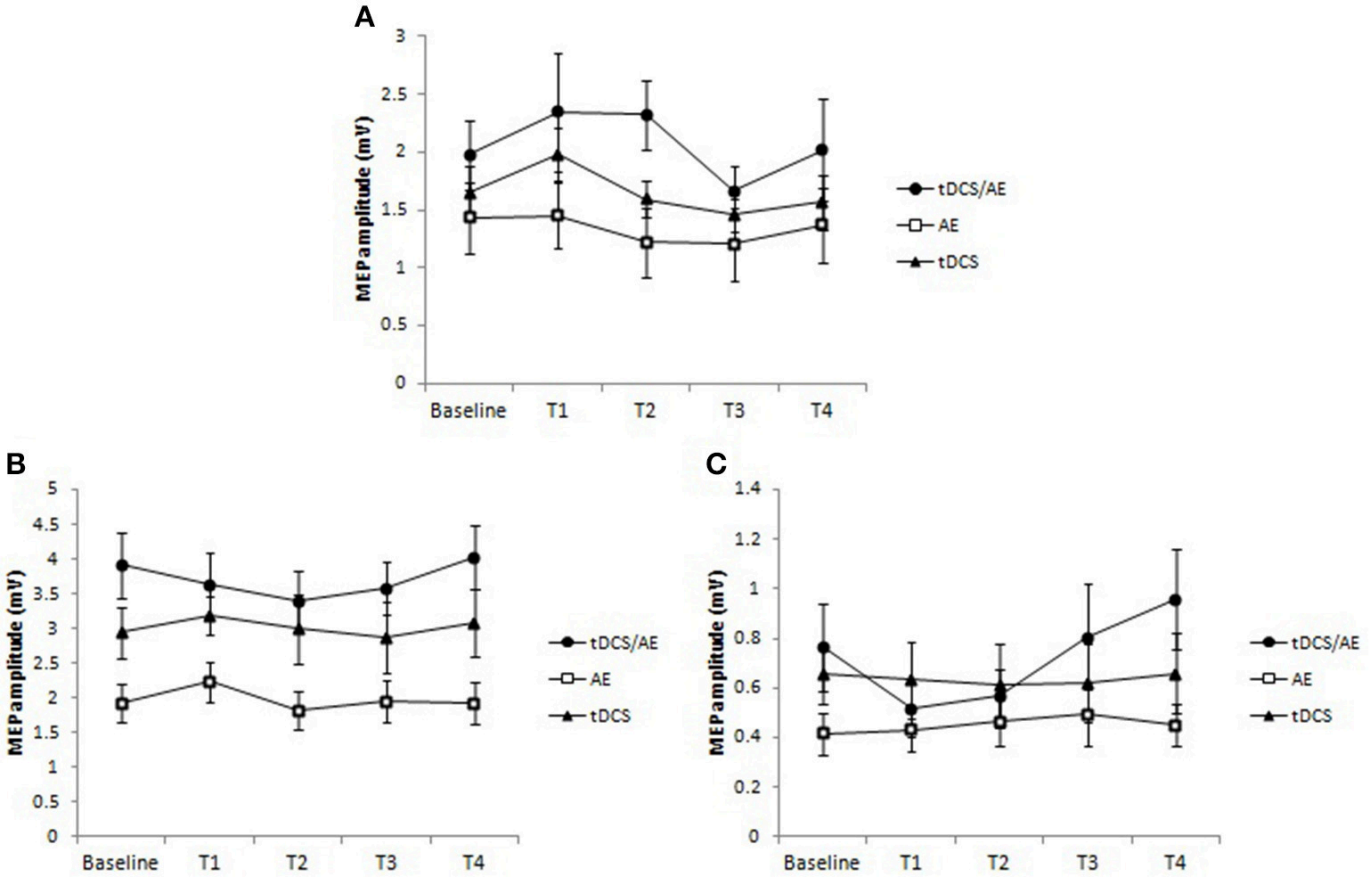
**Data for cortical excitability**. **(A)** Data for motor evoked potential (MEP). There was an increased excitability in the tDCS/AE group until the end of the intervention, not being maintained after the period of follow-up. For tDCS group there was an increase in cortical excitability after just 1 week period of intervention that occurred with active stimulation. The EA group showed a slight decrease in MEP after the intervention. **(B)** Intracortical facilitation. There was a slight decrease in intracortical facilitation, represented by decreased amplitude of MEP's only in group tDCS/AE. **(C)** Intracortical inhibition. There was an increase in intracortical inhibition during the protocol period for tDCS/AE group, and in the follow-up periods an increase above the baseline. No statistically significant findings were observed. Data shown as mean and standard error.

With regard to TMS parameters, there were no significant results for the main analysis of time vs. group for MEP [*F*_(8, 128)_ = 0.57, *p* = 0.8], or time [*F*_(2, 168)_ = 2.34, *p* = 0.057], and group had a significant effect [*F*_(2, 168)_ = 5.37, *p* = 0.005]. These results were similar to those of the other TMS variables. There were no effect of the interaction of time and group for ICF [*F*_(8, 128)_ = 0.56, *p* = 0.8] and for the interaction effect of ICI [*F*_(12, 128)_ = 1.6, *p* = 0.9]. The data for this variable are shown in Figure 7.

#### Regression analysis

To better understand the influence of demographic, clinical outcomes, and also the baseline pain status on the pain response to the interventions, we initially ran univariate regression models, considering the difference between pain scores before and after the treatment as the dependent variable. The independent variables were the baseline values of the following: duration of pain, intensity of pain, anxiety, pain threshold, mood, subscales of quality of life, and cortical excitability values (MEP, ICI, and ICF), and also intervention group (type of intervention). We defined significance as *p* < 0.01 for this initial analysis. A relationship was observed between the response in pain intensity (difference between pain scores before and after treatment) and baseline pain intensity values (*p* = 0.01) and baseline anxiety levels (*p* = 0.01). The regression data are shown in Table 4. Correlation analysis were also carried out for those variables. Results are shown in Table 1, at Supplementary Material.

**Table 4:** **Results for univariate linear regression models**.

	***P*-value**	**B Coefficient**
Age	0.9	0.001
Intervention Group	0.09	−0.63
Pain duration	0.6	0.001
PPT	0.7	0.07
MEP	0.6	0.14
ICF	0.5	0.12
ICI PRE	0.1	0.79
SF36 Physical functioning	0.9	0.002
SF36 Physical role functioning	0.4	−0.008
SF36 Bodily pain	0.8	−0.006
SF36 General health Perceptions	0.6	−0.009
SF36 Vitality	0.2	−0.02
SF36 Social role functioning	0.3	0.01
SF36 Emotional role functioning	0.5	−0.004
BDI	0.4	0.022
VNS Pain baseline	0.01	0.44
VNS Anxiety baseline	0.01	0.24

We then performed multivariate regression analysis, with the difference in pain intensity as the dependent variable and baseline pain level, anxiety and mood scores, and the respective interactions as the independent variables.

The multivariate model with mood and baseline pain levels showed significant results (model *p* = 0.0003). Baseline pain correlated positively with changes in pain after treatment (*b* = 0.53 and *p* = 0.002) and mood scores (*b* = 0.07 and *p* = 0.006), indicating that higher pain and depression scores at baseline were associated with a greater pain response. Baseline pain scores appeared to modify the effect of depression on the response to the interventions, indicating that the association between depression and the response to the interventions weakened with a decrease in baseline pain values (Table 5).

**Table 5:** **Pain intensity data stratified by level of depression**.

	**tDCS/AE group**			**AE group**			**tDCS group**		
	**BDI**	**VNS**	%^*^	**BDI**	**VNS**	%^*^	**BDI**	**VNS**	%^*^
No depression or mild depression	11.8	6.0	26	9.1	6.3	31	11.5	6.3	22
Moderate to severe depression	25.7	7.9	37	29.3	7.2	24	29.9	7.4	20

* *Percentage of improvement from the mean values. BDI, Beck depression inventory. VNS, Visual numeric scale*.

#### Adverse effects

All adverse effects were mild and did not differ between treatment groups (Table 6).

**Table 6:** **Side effects occurrence**.

	**tDCS/AE**	**AE**	**tDCS**	**Total**	*P*-value^*^
**AEROBIC EXERCISE**					
**Mild Muscle Pain**	**4**	**3**	**0**	**7**	**0.1**
**tDCS**					
Headache	3	4	3	10	1.0
Neck Pain	1	2	1	4	1.0
Skull Pain	0	0	0	0	-
Skin Injury	0	0	0	0	-
Tingling	3	4	5	12	0.91
Skin Redness	13	7	11	31	0.1
Somnolence	4	3	5	12	0.91
Concentration Issues	1	0	1	2	1.0
Mood changes	0	0	0	0	-

* *Statistics was performed by fisher exact test*.

## Discussion

This study has demonstrated that neuromodulation with tDCS, in association with aerobic exercise training, in fibromyalgia patients effects greater decreases in pain intensity than the individual techniques. Anxiety levels also improved in the combination therapy group. There was a marginal but significant increase in pain threshold in the combination group compared with tDCS alone. The results for depression were better in the tDCS/AE group vs. the other 2 groups, but did not show significant results in statistic. No significant differences in cortical excitability were observed. Baseline pain and mood scores appeared to be related to the response to these treatments.

The main hypothesis of this study is that the combination of techniques has greater effects in pain intensity in patients with fibromyalgia. Previous studies in neuromodulation for fibromyalgia and aerobic exercise have shown that these techniques yield significant results compared with control interventions and baseline symptoms (Marlow et al., 2013; García-Hermoso et al., 2014; O'Connell et al., 2014; Vural et al., 2014). It is important to underscore that a previous review study concludes that there are no positive results for tDCS when considering the aggregate results for different types of chronic pain, particularly in the long term effects (O'Connell et al., 2014). However in this study we chose to evaluate if there is an additive effect when using two modulating techniques.

In this study, the effect of the combination of techniques was compared between two active techniques and the combination of each individual active intervention and the associated placebo method. Although some of the results for the secondary outcomes were marginally significant, this trial was not powered for the secondary outcomes; also, we compared the combined treatment against each group using one active treatment alone.

Aerobic exercise acts systemically in the body, influencing many domains. For instance, it can alter brain activity through motor cortex activation and neurotransmitter release (Meeusen and De Meirleir, 1995). This concept is known as exercise-induced hypoalgesia, which is regulated by the release of endogenous opioids (Koltyn, 2000). In addition, exercise modifies the activity in certain regions in the cortex through facilitatory and learning mechanisms, leading to long-term potentiation (LTP) mechanisms (Erickson and Kramer, 2009; Lojovich, 2010). Various studies have reported beneficial results of exercise in many chronic pain syndromes (Nijs et al., 2012).

However, the results are somewhat mixed. Patients with widespread pain experience immediate worsening of symptoms due to dysfunction in endogenous analgesia mechanisms, which might be related to myofibril injury, which causes inflammation and increased nociceptive signaling. Therefore, its use is somewhat limited. To obtain beneficial results it is necessary to overcome this phase, which is not achieved by a majority of patients.

Another method of influencing the motor system is neuromodulation with tDCS (Mendonca et al., 2011). The basic mechanism of action of tDCS is modulation of spontaneous neuronal firing through induced polarization of neural tissue. In this context, anodal tDCS leads to depolarization and thus an increase in spontaneous neuronal firing; cathodal tDCS has the opposite effects. tDCS effects motor cortex activation (M1), resulting in secondary modulation of regions that are associated with pain modulation (Castillo Saavedra et al., 2014).

Continued use of tDCS induces plastic changes and can lead to pain relief for 1 month after the end of the intervention (Fregni et al., 2006). Several studies have shown that five consecutive applications of tDCS over the M1 relieve pain and improve quality of life and sleep in various chronic pain syndromes (Fregni et al., 2006; Roizenblatt et al., 2007; Valle et al., 2009).

Based on the effects of aerobic exercise and the mechanisms of tDCS, we hypothesized that the combined therapy would be more effective than each method alone, because tDCS would prime the system, which would be subsequently modified by aerobic exercise. Another possibility is that these techniques have disparate neural targets and thus do not synergize. Our data on the neurophysiological assessment with TMS, demonstrating no changes in cortical plasticity of the motor cortex between the interventions, supporting that the additional effects of the combination therapy are related to the activity in other neural circuits, independent of the motor system.

Other protocols that have testing the combination of tDCS with other techniques have been reported. Riberto et al. (2011) developed a regimen, comprising tDCS and a multidisciplinary rehabilitation program, observing improvement in only one variable in the SF-36 questionnaire for quality of life (pain domain only). The authors implemented an exercise program, which used stretching and ergonomic and posture instructions 3 times per week, and performed tDCS once per week for 10 weeks. The difference in the use of tDCS might explain the differing results comparing to our results—achieving long-term effects requires a protocol with more days of stimulation in a shorter time (Nitsche et al., 2008).

Another study (Boggio et al., 2009) showed that a single application of tDCS, associated with the use of peripheral TENS, for the treatment of chronic pain had a superior effect compared with tDCS or TENS alone. For other areas, such as cognitive and motor rehabilitation, several trials have combined tDCS with various training methods, including robotics, virtual reality, and computer-based training (Soler et al., 2010; Giacobbe et al., 2013; Martin et al., 2014), the results of which support combination treatment to enhance and guide the effects of tDCS.

We evaluated cortical excitability using single-pulse and paired-pulse TMS to assess motor cortex plasticity changes that were associated with these three groups of treatments. A previous study demonstrated that FM subjects experience alterations in these parameters, such as increased MEPs at rest and reduced ICI and ICF (Salerno et al., 2000; Mhalla et al., 2010). Although, our results were marginally significant, we noted an overall increase in MEP, a decline in ICI, and small changes in ICF. But, we did not observe any significant differences between groups. In contrast, Antal et al. (2010) observed a reduction in ICI after anodal stimulation, but they used different stimulation parameters (smaller electrode size and intensity of 1 mA). In a study using repetitive TMS (rTMS) using 10 Hz of intensity, which also activates the motor cortex, the authors demonstrated an increase in ICI in accordance to our results (Lefaucheur et al., 2006; Dall'Agnol et al., 2014). The lack of difference between groups but the disparate behavioral results suggest that the differential results are attributed to nonmotor neural circuits.

Notably, the initial level of pain and mood appears to be a predictor of outcome. We observed that individuals with higher pain levels and higher levels of depression responded better to the treatment, indicating greater central sensitization that might be responsive to the combined intervention; however the results of the prediction model need to be interpreted carefully given the relatively small sample size for this analysis.

A limitation of this research is related to blinding. There is a debate on the effectiveness of blinding in tDCS studies. Some studies such as the one from Villamar et al. (2013) shows that blinding in single session cross-over studies is not adequate. However, further studies such as the one from Brunoni et al. (2014) showed that blinding in clinical trials in which the treatment effect plays a major role, such as the blinding method of tDCS is comparable to drugs such as sertraline (Brunoni et al., 2014). Regardless we did not conduct blinding assessment given the questionable results of this assessment as patients may correlate stimulation condition with improvement. Finally, we observed that there were no significant differences in adverse effects of tDCS, not even when with regard of skin redness (86% of individuals in the tDCS/AE group, 47% of individuals in the EA group, 73% of individuals in the tDCS group, without significant results *p* = 0.1) shown in Table 4. There were also no differences in adverse effects of aerobic exercise. In addition, all groups received two forms of intervention associated, with one active intervention at least which may have also helped to maintain blinding of the other intervention. Another limitation is related to loss of follow-up. Although this is a potential source of bias, missing data were completely at random and distributed equally across groups of treatment. Also it is important to highlight that all patients completed the entire month of intervention and the subsequent final evaluation. In addition, sensitivity analysis showed no significant differences in the results.

Based on these findings, the three groups showed positive effects in many variables, such as pain relief, quality of life, depression, and anxiety, but there was a larger effect that was associated with the combination treatment. The simultaneous effect of the combination treatment on pain and depression levels in fibromyalgia should prompt larger trials on the effects of this modality with longer follow-up periods.

## Author contributions

MM contributed with project creation, data collection, statistical analysis and article writing. MS contributed with data collection, statistical analysis and article writing. LG contribute with data collection, statistical analysis and article writing. LB contributed with article writing. AB contributed with project creation and article writing. FF contributed with project creation, data collection, statistical analysis and article writing.

### Conflict of interest statement

The authors declare that the research was conducted in the absence of any commercial or financial relationships that could be construed as a potential conflict of interest.
