# Supplementary material for: Transcranial Direct Current Stimulation Combined with Aerobic Exercise to Optimize Analgesic Responses in Fibromyalgia: A Randomized Placebo-Controlled Clinical Trial
Source: Front Hum Neurosci. 2016 Mar 10;10:68. doi: 10.3389/fnhum.2016.00068 (PMC4785149; doi:10.3389/fnhum.2016.00068)
Supplement: Supplementary file 1 [file Table1.docx]

| **Supplementary Table 1 – Results for Pearson Correlation** | | |
| --- | --- | --- |
|  | P value | Coefficient |
| Age | 0.95 | -0.009 |
| Intervention Group | 0.09 | 0.25 |
| Pain duration | 0.6 | -0.07 |
| PPT | 0.7 | -0.04 |
| MEP | 0.6 | -0.07 |
| ICF | 0.5 | -0.09 |
| ICI | 0.18 | -0.19 |
| SF36 Physical Functioning | 0.9 | -0.01 |
| SF36 Physical Role Functioning | 0.4 | 0.11 |
| SF36 Bodily Pain | 0.7 | 0.03 |
| SF36 General Health Perceptions | 0.6 | 0.07 |
| SF36 Vitality | 0.22 | 0.18 |
| SF36 Social Role Functioning | 0.34 | -0.14 |
| SF36 Emotional Role Functioning | 0.5 | 0.08 |
| BDI | 0.4 | -0.11 |
| VNS Pain baseline | 0.01 | -0.36 |
| VNS Anxiety baseline | 0.01 | -0.36 |
